# Optimising digital advance care planning implementation in palliative and end-of-life care: a multi-phase mixed-methods national research programme and recommendations

**DOI:** 10.1186/s12916-025-04114-x

**Published:** 2025-05-20

**Authors:** Matthew J. Allsop, Jacqueline Birtwistle, Michael I. Bennett, Andy Bradshaw, Paul Carder, Catherine J. Evans, Robbie Foy, Ciara Heavin, Barbara Hibbert, Pablo Millares Martin, Sam D. Relton, Suzanne H. Richards, Maureen Twiddy, Katherine E. Sleeman

**Affiliations:** 1 Leeds Institute of Health Sciences, University of Leeds, Leeds, UK; 2 Cicely Saunders Institute of Palliative Care, Policy & Rehabilitation, King’s College London, London, UK; 3 NHS West Yorkshire Integrated Care Board, Wakefield, UK; 4 Cork University Business School, University College Cork, Cork, Ireland; 5 Whitehall Surgery, Leeds, UK; 6 Institute of Clinical and Applied Health Research, Hull York Medical School, University of Hull, Hull, UK

**Keywords:** Digital health, Palliative care, Care coordination, Advance care planning

## Abstract

**Background:**

Digital advance care planning (DACP) is increasingly used globally for patients with life-limiting conditions to support real-time documentation and the sharing of preferences for care. There has been low engagement with DACP systems, with patients often having information about their care preferences documented late in their illness trajectory or not at all. To optimise implementation, the Optimal Care research programme sought to understand DACP system use from multiple perspectives to guide their development and evaluation.

**Methods:**

Between 2020 and 2023, our mixed-methods research programme sought an understanding of DACP implementation from multiple perspectives, including (i) national online survey of end-of-life care commissioning leads in England; (ii) online survey of community and hospital-based health and care professionals in two geographical regions; (iii) semi-structured interviews with a sample of survey respondents; (iv) focus groups and interviews with patients with life-limiting illness and their carers and (v) regional and national Theory of Change workshops. Findings were organised by five phases of a conceptual model of DACP generated during the programme and further categorised using the Non-adoption, Abandonment, Scale-up, Spread and Sustainability (NASSS) framework.

**Results:**

A total of 788 stakeholders participated. Twenty evidence-based recommendations were distilled from data collected across the research programme to guide the implementation of DACP in routine care. Considerations are provided across the five phases of DACP implementation (system design, recognition of clinical need for DACP, documentation processes, health and care professional engagement with DACP and DACP evaluation). Recommendations prioritise a focus on end-user needs and experiences, alongside highlighting the requisite need for DACP systems to support information exchange across settings involved in the care of people with life-limiting conditions.

**Conclusions:**

As currently designed and implemented, DACP systems may be falling short of their potential and are not working as intended for patients, carers and health and care professionals. The application of the recommendations should ensure consideration of the wider ecosystem in which DACP is being implemented, prioritising end-user experiences. Future research should prioritise developing approaches that target health and care professional DACP system engagement, alongside developing and evaluating patient and carer access to DACP systems.

## Background

Patients with life-limiting illnesses or complex needs should be offered the opportunity to take an active role in planning for their future health and care [1–3]. This is consistent with the principle of patient-centred care as a collaboration between patients, families and healthcare providers, to guide decisions aligned with patient preferences and needs [4]. Advance care planning is a process that ‘enables individuals to identify their values, to reflect upon the meanings and consequences of serious illness scenarios, to define goals and preferences for future medical treatment and care, and to discuss these with family members and health and care providers’ [5]. Patient preferences can change over time, therefore, information on preferences should be up-to-date and readily accessible by health and care providers at the point of need [3]. Internationally, advance care planning is increasingly recognised and encouraged and is established practice in the UK [3], USA [6, 7], Canada [8], Australia [9], New Zealand [10] and parts of Europe (e.g. [11–13]).

Patients are most likely to experience care that aligns with their preferences when key information is elicited in a quality discussion, documented in a standardised format, shared across health services in real time and accessed by health and care professionals [5, 14]. Increasingly, digital systems are being used to support this process through real-time documentation of advance care planning discussions [15, 17, 18]. Digital approaches may capture varying elements of advance care planning information, including advance statements of wishes and preferences, advance decisions to refuse treatment (e.g. cardiopulmonary resuscitation, antibiotics) and details of lasting power of attorney [19]. Such systems are being developed and implemented internationally, including in the USA [20, 21], Australia [22, 23], and the United Kingdom (UK) [18, 24].

Despite their increasing availability, there has been low engagement with digital advance care planning (DACP) approaches internationally, with patients who would benefit often having this information documented late in their illness trajectory or not at all [20, 25]. The development and implementation of DACP has also been mostly pragmatic, with a lack of empirical research on their development and implementation [18]. To date, there has been limited exploration of the perspectives of those involved with DACP, including patients and carers, to determine how the use of DACP can be optimised [26, 27]. The Optimal Care study (2020–2023) sought to generate evidence to guide the development and evaluation of DACP systems. The objectives were to examine the implementation processes of DACP systems; to understand how health and care professionals, patients and carers engage with and experience DACP systems; and to identify best practice for implementation, evaluation methods and strategies to enhance engagement. This paper synthesises findings across all workstreams and presents recommendations for DACP system implementation.

## Methods

### Design

**Fig. 1:**
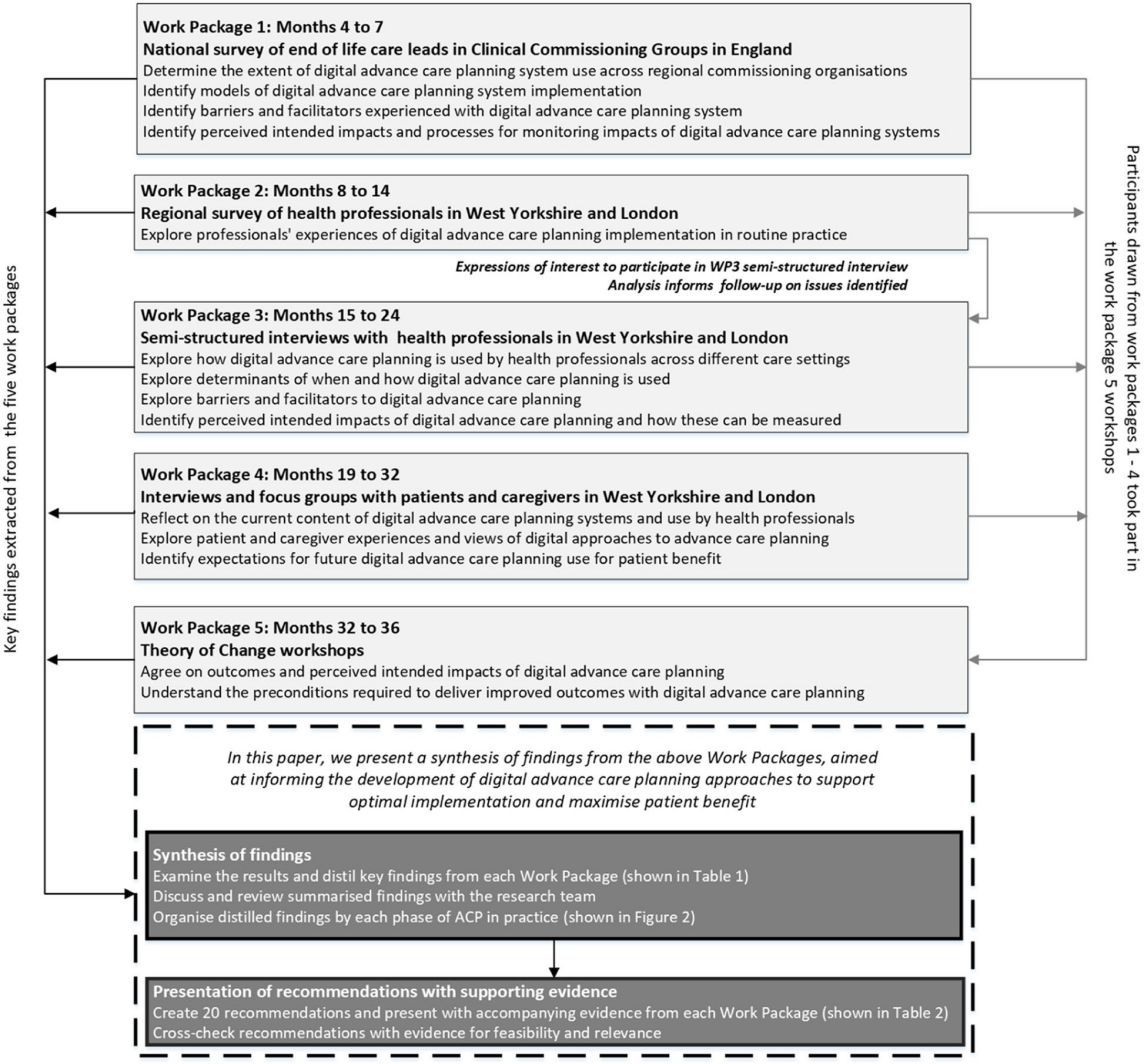
Schematic outlining the five work packages of the optimal care study and the synthesis of findings to generate recommendations for digital advance care planning implementation

This research programme adopted a multi-phase mixed-methods design [28] and comprised five sequential Work Packages (WPs) (Fig. 1). The research programme was conducted in England between December 2020 and November 2023, where DACP systems have been in development since 2008 [1].

In the UK National Health Service (NHS), information technology systems are funded through multiple sources, including central government (i.e. the Department of Health and Social Care), local NHS trusts (semi-autonomous organisational units within the NHS generally serving a geographical area or a specialised function) and through public–private collaborations. Current national end-of-life care policy promotes digital systems to support the documentation, storage and sharing of patient preferences for care [3]. This aligns with calls to integrate information technologies in ways that enhance patient experiences [29] and improve care quality and coordination for people receiving palliative and end-of-life care [2].

DACP systems in England are commonly referred to using the term Electronic Palliative Care Coordination Systems (EPaCCS) [15, 30]. EPaCCS have been implemented with different configurations across England. For example, one of the earliest EPaCCS systems used in London, ‘Coordinate my Care’, was a standalone web-based electronic register [31]. Approaches used in other parts of England include having a template within existing electronic patient record systems used across settings. For example, in Leeds, a city in West Yorkshire, records are created within an electronic record system used by general practitioners in primary care and hospice teams [25]. In this way, the electronic patient record serves as a key source of health data for direct patient care across various health and social care organisations [32]. Information standards published in 2022 seek to standardise the content of EPaCCS, irrespective of the platform used [33]. While the term EPaCCS is used in England, throughout this paper we use the more internationally recognised term digital advance care planning (DACP).

**Fig. 2:**
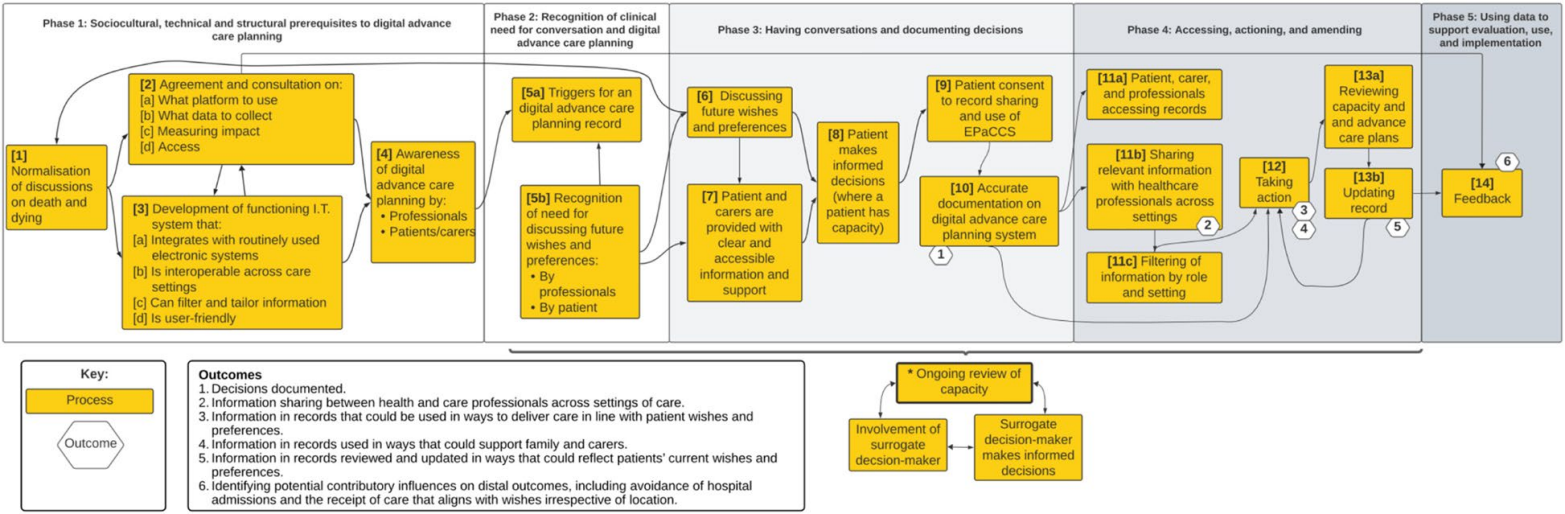
Conceptual model depicting the interventions and outcomes influencing digital advance care planning implementation, as reported in [40]

The five sequential WPs undertaken across the programme included:

- WP1: A national cross-sectional online survey of end-of-life care commissioning leads (*n* = 85) in England. Leads in each commissioning area were emailed a secure link to the survey. Survey items were informed by an earlier systematic review by team members [18] and earlier survey of DACP use in England [34]. Leads came from a range of backgrounds (e.g. healthcare management, and clinical backgrounds across different community and hospital-based care settings). We enquired about the current implementation status of DACP systems, their role in information sharing and intended impact, and requested routine patient-level data relating to DACP [30].
- WP2: A cross-sectional online survey of professionals (*n* = 569) across community and hospital settings in West Yorkshire (*n* = 189; 33.2%) and London (*n* = 380; 66.8%). West Yorkshire and London were selected to enable the exploration of professionals, patients and carers experiences across settings in which multiple approaches to DACP implementation have been used. At the start of the project, West Yorkshire had ten different DACP systems, each having been launched at different times. In London, Coordinate My Care [35] was a single platform used across the region. Engaging participants in the two regions enabled the exploration of varied DACP implementation models. We purposively sampled healthcare professionals in care settings working with patients with progressive chronic diseases or palliative care needs. The survey included items adapted from the Normalisation MeAsure Development questionnaire (NoMAD) implementation measure [36] to explore healthcare professionals’ perceptions of the value and impact of DACP.
- WP3: An interview study with purposively sampled healthcare and social care practitioner respondents (*n* = 52) from the WP2 survey to include representation from different care settings (ambulance, community nursing, hospital palliative care, general practice, care homes and community specialist palliative care) and regions (i.e. West Yorkshire and London). Individual online semi-structured interviews, informed by Normalisation Process Theory [37], were conducted to explore the factors that influence the implementation of DACP systems in routine clinical practice across different care services and settings in West Yorkshire and London [38].
- WP4: Eight focus groups and fifteen semi-structured interviews with a purposive sample of patients (*n* = 29) and current or bereaved carers (*n* = 15) in London and West Yorkshire [39]. Participants were recruited from hospice settings, non-governmental support and advocacy groups and care home residents. Interviews and focus groups explored participants’ perspectives on DACP, the anticipated impact from their use, and expectations for their future development.
- WP5: An exploratory qualitative study incorporating three Theory of Change workshops [40] with participants (*n* = 38). This included two in-person workshops (in London (*n* = 16) and West Yorkshire (*n* = 14)), and one online workshop that included a varied sample (*n* = 8) comprising patients, carers and health and care professionals (including those with commissioning responsibilities). The workshops informed the development of a conceptual model that depicted how DACP systems are currently being implemented in routine care and factors to consider around their implementation (Fig. 2). We also reported on differences and uncertainty among patients, carers, professionals and participants with commissioning responsibilities relating to what DACP systems are, who they are for, their purpose and how they should be evaluated.

### Synthesis

From the outset of the programme, we planned to integrate findings from all five WPs to guide how DACP approaches could be optimally implemented. The synthesis of findings and generation of recommendations was undertaken through the following steps:

1. The research team reviewed the analyses and outputs from the five WPs, deriving key findings to guide and inform the development of DACP approaches.
2. Team members (JB, MA) conducted a deductive framework analysis, aligning key findings from WPs 1–4 with the five phases of the conceptual model of DACP implementation generated during WP5 (Fig. 2) [40]. These were as follows: (i) sociocultural, technical and structural prerequisites; (ii) recognition of the clinical need for conversation; (iii) having conversations and documenting decisions; (iv) accessing, actioning and amending; and (v) using data to support evaluation, use and implementation.
3. The Non-adoption, Abandonment, Scale-up, Spread and Sustainability (NASSS) framework [41] domains were used to organise findings within each of the five phases where relevant data were present. These domains include the condition, the technology, the value proposition, the adopter system (comprising professional staff, patient and lay carers), the organisation(s), the wider (institutional and societal) context and sustainability and adaptation over time.
4. Finally, through a series of research team discussions, recommendations were organised in related groupings, followed by the generation of each recommendation underpinned by programme findings. The recommendations were discussed and further refined across the team. A final table was agreed upon that included the derived recommendations alongside the supporting findings from across the research programme.

### Ethics

WP1 obtained approval from the North of Scotland Research Ethics Committee (research ethics committee reference, 21/NS/0046). WPs 2–5 obtained approval from the North of Scotland Research Ethics Committee (21/PR/0428). All participants gave their written informed consent to participate.

## Results

In total, 788 stakeholders contributed across the five WPs. Table 1 outlines the characteristics of participants and summarises the key findings from each respective WP.

**Table 1:** Summary of key findings derived from the five work packages of the optimal care research programme

Work package (WP)	Summary of key findings
WP1: A national cross-sectional online survey of end-of-life care commissioning leads Respondents (*n* = 85) in WP1 included regional leads for commissioning in palliative and end-of-life care in England	• The majority of respondents (67.1%) stated there was an operational DACP system in place in the region for which they were responsible for commissioning. The remaining regions were either planning to implement a DACP system (15.3%) or had no plans in place to implement one (17.6%) • While most DACP systems were designed to support information exchange across care settings, there were often critical gaps. Most DACP systems were not accessible to staff in care homes or ambulance trusts • The rationale for having a DACP system often aligned with national policy goals, including having DACP systems to enhance coordination of care, support early identification and recording of people approaching the end of life and reduce avoidable hospital admissions [42] • Over half of respondents did not monitor the impact of DACP systems on care delivery. Some respondents reported using proxy measures (such as a person dying in their preferred place of death) that aligned poorly with the intended impact of DACP approaches. Few respondents reported collection of patient, caregiver or healthcare professional feedback, or detailed case studies, to assess the intended impact of DACP systems • Common implementation challenges for DACP systems included a lack of interoperability within existing digital infrastructure and a lack of key stakeholder engagement, such as general practitioners and other clinical staff • Using data submitted by respondents relating to their commissioning regions, it was determined that overall one-third of patients had a record created on a DACP system prior to death. Where a DACP record had been created, fewer than one-third of the records contained key information relating to care preferences of the person (e.g. preferences around place of death). Between April 2019 and March 2020, a median of 33.22% of people had a DACP record, increasing slightly to 42.91% between April 2020 and September 2020 • Nearly half of patients with a DACP system record had a diagnosis of cancer. Participants reported an increase in the initiation of records following the start of the Coronavirus disease 2019 (COVID-19) pandemic *A comprehensive overview of WP1 findings has been published elsewhere* [30]
WP2: A cross-sectional online survey of health and care professionals across both community and hospital settings Respondents (*n* = 569) included health professionals, including those from West Yorkshire (*n* = 189; 33.2%) and London (*n* = 380; 66.8%). The largest proportion of respondents came from general practice teams (*n* = 254; 44.6%) and included general practitioners, practice nurses and allied health professionals. Respondents included those from hospice teams (85;15.0%), ambulance workers (68; 12.0%), care homes (64; 11.2%), hospital teams including doctors, nurses and allied health professionals (50; 8.8%) and community nurses (48; 8.4%)	• There was variation in the way DACP systems are perceived and used across the two geographical regions surveyed, and among professionals working in different settings involved in palliative care delivery • Professionals in London were more likely to report being familiar with DACP systems. However, in West Yorkshire, participants rated DACP systems more favourably, viewing them as a legitimate part of their role and emphasising collaborative efforts to appraise the systems and their components • In both regions, there was low to moderate agreement on whether DACP systems were worthwhile and of value for respondents from ambulance teams, community nursing teams, hospital teams and general practice teams. Respondents from both hospice and care home teams were most likely to view DACP systems as worthwhile and of value • There was broad agreement that DACP systems did not disrupt working relationships. However, many healthcare professionals expressed ambivalence regarding their colleagues’ skills and confidence in effectively using DACP systems • DACP systems were viewed as supporting people to communicate advance care planning information across NHS settings, but there was less certainty about their ability to share information with social care services • A lack of access to electronic devices, training and knowledge relating to advance care plans were common barriers. These experiences may have influenced perceptions of the ability of these systems to share information • Alternative approaches to documenting advance care plans were reported. DACP systems were often running in parallel with similar advance care planning initiatives using both paper and digital formats *A comprehensive overview of WP2 findings has been published elsewhere* [43]
WP3: A qualitative interview study with health and social care professionals Participants (*n* = 52) included health and social care professionals from London (*n* = 29) and West Yorkshire (*n* = 23). Participants including a range of professionals working across the settings of primary care (12;23.1%), hospice (12; 23.1%), care homes (9;17.3%), hospitals (8;15.4%), ambulance teams (6;11.5%) and community nursing teams (5;9.6%)	• Findings from semi-structured interviews with health and social care professionals suggest that DACP systems are not working as intended for facilitating person-centred care • Professionals lacked confidence that the quality of the information recorded in DACP systems aligned with patient wishes. Professionals often preferred to speak directly with a health professional who had recently seen a patient to confirm the validity of advance care planning information, sometimes bypassing the record entirely • A key issue was technological limitations, with separate DACP records stored across parallel patient electronic record systems or failing to provide sufficient documentation or access • Professionals’ experiences resonated with previously documented challenges relating to advance care planning such as lack of time and lack of care continuity. [44–46] Lack of clarity over which professionals should contribute to recording information and the timing of these contributions often resulted in poor-quality data • Care home staff reported use of local electronic record systems to document care preferences and wishes of residents, but the information contained within them was mostly inaccessible to external services. Care homes were also unable to access information from DACP systems used by NHS services *A comprehensive overview of WP3 findings has been published elsewhere* [37]
WP4: A qualitative study with patients and current or bereaved carers Participants (*n* = 44) included patients and current or bereaved carers from London (*n* = 15/*n* = 10, respectively) and West Yorkshire (*n* = 14/*n* = 5, respectively). Patient participants included those living with cancer (*n* = 11) and non-cancer conditions (*n* = 18), with varying levels of experience of having wishes and preferences documented on a digital system. Patient and carer participants varied across age groups, with majority of participants (*n* = 38;86.4%) reporting White British as their ethnicity, alongside Asian (*n* = 4;9.0%), Black Caribbean (*n* = 1;2.3%) and Mixed or multiple (*n* = 1;2.3%)	• Semi-structured interviews and focus groups with patients and their carers highlighted requirements for DACP systems • Patients’ levels of engagement with advance care planning discussions and subsequent documentation of wishes and preferences were varied. Some participants actively pursued advance care planning and associated documentation via online resources, while others were unaware that this was available or indicated that it was not wanted • Prior experiences involving digital health records influenced participants’ views on how DACP would support their future care. Situations where health information had not been shared between services when expected, and instances of incorrect information about patients being recorded, led to concerns around the accuracy of advance care planning information, and not knowing what information, if any, was already held about patients and who could access and use it • Skilled discussions are needed to support advance care planning decision-making and documentation. It was often unclear which health professional could be approached to support this discussion, and getting a face-to-face appointment with a general practitioner for this type of discussion was perceived to be difficult • Participants valued potential impacts of DACP such as improved outcomes relating to upholding personal autonomy and emotional, social and spiritual factors *A comprehensive overview of WP4 findings has been published elsewhere* [39]
WP5: An exploratory qualitative study incorporating Theory of Change workshops Participants included 38 participants (16 in a London workshop, 14 in a West Yorkshire workshop, and 8 in an online workshop) including patients, carers and health and care professionals (including those with commissioning responsibilities). Patient participants (*n* = 4;10.5%) including those with cancer (*n* = 2;5.3%) and multiple long-term conditions (*n* = 2;5.3%)	• A conceptual model depicting the key phases of DACP use in practice was developed [47]. The development of the model drew upon the NASSS framework which highlights how multiple influences (e.g. the technology, organisation(s) and wider system) may influence DACP use. The resulting conceptual model included five distinct phases relating to DACP use in practice: 1. Sociocultural, technical and structural prerequisites (e.g. functioning IT systems across care settings, awareness and use of systems by professionals) 2. Recognition of the clinical need for an advance care planning conversation and its digital documentation 3. Having conversations and documenting decisions 4. Accessing, actioning and amending digital advance care plans 5. Using data to support evaluation, use and implementation of digital advance care planning systems • The workshops facilitated the capture of varied views to enrich an understanding of current DACP system implementation, including factors relating to the technology (e.g. IT systems used to access, store and share information) and human factors (e.g. roles and responsibilities relating to accessing and using digital advance care planning systems) • There were differences and uncertainty across patients, carers, health and care professionals and commissioners, relating to what DACP systems are, who they are for and how organisations should evaluate them • Participants across professional groups understood the value of DACP systems primarily in terms of service delivery and clinical decision-making, including achieving the preferred place of care and death, reflecting outcomes typically reported in research and policy. [18] • Patients and carers considered additional information that could be captured and shared through DACP approaches, beyond what is currently collected. This included broader aspects of care relating to symptom management, preferred and unwanted medical interventions, family and carer support (including through bereavement) and maintaining social ties • Process-related outcomes that may be straightforward to quantify were identified (e.g. if a DACP record is documented or updated, and how many times a record is accessed). However, the data demonstrated a gap in understanding how DACP systems may influence broader outcomes such as care quality (e.g. achieving a preferred place of care or death, delivering care in line with wishes and preferences, avoidance of hospital admissions) *A comprehensive overview of WP5 findings has been published elsewhere* [40]

A series of twenty recommendations to improve the design and implementation of DACP systems were generated, organised according to the five phases of the conceptual model (Fig. 2) and the NASSS framework (see Table 2). In Appendix 1, we provide a more detailed overview of the twenty recommendations and the rationale and supporting evidence underpinning recommendations generated throughout the research programme.

**Table 2:** Optimal Care recommendations to improve the design and implementation of digital advance care planning systems aligned with five phases of DACP and the domains of the NASSS framework

Phase of DACP implementation	Recommendations generated from the Optimal Care research programme	Alignment with NASSS framework domain
Phase 1: Sociocultural, technical and structural prerequisites	Recommendation 1. DACP systems should accommodate patient diversity—including varying disease trajectories and patient and carer backgrounds	*Domain 1 - Health condition.*
	Recommendation 2. Engagement with key stakeholders (i.e. health and care professionals, patients and carers) is essential to guide the content of DACP systems, alongside ensuring expectations, requirements, procedures and impact of the documentation and sharing of digital advance care plans are clear.	*Domain 2 - Technology*
	Recommendation 3. Organisations should prioritise and build DACP systems using platforms that support interconnectivity. This should ensure authorised health and social care workers across all relevant care settings can access digitally stored advance care planning documentation.	*Domain 2 - Technology*
	Recommendation 4. Agreement on the purpose and intended impact of DACP systems should be established through consultation with all stakeholders involved in the care of patients with life-limiting illnesses, alongside patients and carers themselves. This engagement should guide the selection and measurement of DACP outcomes, which must be developed locally to account for geographical variations and differing approaches to DACP.	*Domain 3 - Value proposition*
	Recommendation 5. The limitations of sharing information between services and regions should be clear to health professionals should this need to be communicated to patients.	*Domain 4 - Adopters*
	Recommendation 6. Organisations should learn from others who have developed activities to promote uptake and use of DACP systems (such as strengthening engagement or leadership and development of training). There is scope to identify and learn from creative solutions to promote uptake and use.	*Domain 5 - Organisations*
	Recommendation 7. Organisations should consider ways of embedding DACP into routine structures and processes (e.g. at admission, at discharge, multidisciplinary team meetings and handovers).	*Domain 5 - Organisations*
	Recommendation 8. Training delivered within organisations should include data protection and legal or regulatory implications of methods of planning future care.	*Domain 6 - Wider System*
	Recommendation 9. Initiatives that empower patients and families to engage in discussions about death and planning for end-of-life issues should be pursued. This may include the circulation of accessible resources (pamphlets, videos, website material) that offer guidance, alongside public engagement events such as Dying Matters Week.	*Domain 6 - Wider System*
Phase 2: Recognition of the clinical need for conversation and	Recommendation 10. Patients should be identified and offered the opportunity to document a digital advance care plan irrespective of disease type, with approaches explored to support the identification of non-cancer conditions.	*Domain 1 - Health condition*
	Recommendation 11. There is scope to leverage existing community-based assets including organisations that provide in-person and patient-facing resources designed to support people to develop their own advance care plans. Existing patient-facing resources (including online resources) can be used to support preparation and readiness for advance care planning discussions.	*Domain 4 - Adopters*
Phase 3: Having conversations and documenting decisions	Recommendation 12. Efforts should be made to ensure stored information is accessible through existing electronic medical record systems, for example, summary care records in England. Attempts should be made to ensure synchrony of information across record systems to ensure professionals can access exact and up-to-date patient preferences.	*Domain 2 - Technology*
	Recommendation 13. Health professionals should elicit information from patients regarding any paper or electronic self-completed advance care plans they may hold and consider how to merge this information with the organisation’s digital system.	*Domain 4 - Adopters*
	Recommendation 14. Undertaking a needs assessment of levels of staff competence in conducting advance care planning conversations with patients with life-limiting conditions can identify opportunities for delivering and tailoring training. There may also be scope to link competencies around communication and advance care planning within job descriptions for health and care professionals.	*Domain 5 - Organisations*
	Recommendation 15. There is a need for agreement and clarity on which services and settings can interact with a digitally stored advance care plan as soon as it is ready to share. This includes who can review, update and use the information to inform decision-making.	*Domain 5 - Organisations*
	Recommendation 16. Processes should be explored to ensure the content of DACP system records remain up-to-date and accurate. These processes may include, for example, options for patient and carer access to review the content of records or through discussion with patients. Different patients have varying preferences and comfort levels with technology, so access should allow flexibility with access and security (e.g. using either biometrics, security keys, social media credentials or passwords).	*Domain 5 - Organisations*
Phase 4: Accessing, amending, actioning	Recommendation 17. Health professionals must ensure the advance care planning information they record is sufficient, but presentation in a summary or succinct form (e.g. highlighting resuscitation preference) may ensure accessibility and utility for different professional groups enabling them to provide care that aligns with patient wishes.	*Domain 2 - Technology*
	Recommendation 18. Electronic patient record system notifications should flag patients with a digitally recorded advance care plan, and prompt clinicians to access and review. This should be across settings, including options for health and social care professionals working in relevant acute hospital specialties (e.g. emergency department, oncology, respiratory, cardiology) to have viewing access to DACP records created externally to the care setting at a minimum.	*Domain 2 - Technology*
	Recommendation 19. While interacting with the digital systems, health professionals should work alongside patients (and their carers, if the patient permits) by offering to share the screen so patients feel part of their own planning.	*Domain 4 - Adopters*
Phase 5: Using data to support evaluation and implementation	Recommendation 20. The processes associated with the implementation of DACP systems and their effect on patient care should be measured and findings should be reported back to health professionals as well as organisation management. A range of appropriate methods of measurement may be needed to capture how DACP systems influence patient care and clinical decision making.	*Domain 2 - Technology*

### Phase 1: Sociocultural, technical and structural prerequisites (Recommendations 1–9)

Recommendations derived for this phase describe aspects that need to be considered and in place ahead of a DACP system implementation and use as part of routine care. To ensure patients with complex health conditions receive optimal support, organisations should consider implementing DACP systems across a wider range of medical specialities rather than limiting this to palliative care teams. Engagement with health professionals and patients is essential for shaping system content and ensuring all stakeholders have a clear understanding of documentation, procedures and potential impacts of DACP. Organisations should procure software that enables seamless interconnectivity across health and social care, providing healthcare workers with easy access to advance care planning documentation. Those seeking to implement a new system can benefit from the experiences of others with a track record of adopting innovative practices to enhance uptake and engagement. Establishing agreement on the purpose and intended outcomes of DACP requires collaboration among all stakeholders, including patients and carers. Engagement of patients in advance care planning discussions within structured clinical interactions—such as during an initial appointment, hospital admission, discharge or multidisciplinary meetings—can support the embedding of DACP processes into routine care. Any inter-regional or inter-service limitations in data sharing should be clearly communicated to health professionals. Furthermore, fostering public awareness about end-of-life planning is key. Non-governmental organisations (e.g. Age UK and Macmillan Cancer Support) continue to play a role by offering resources or hosting events that introduce patients to advance care planning concepts, fostering greater awareness and involvement.

### Phase 2: Recognition of the clinical need for conversation and digital advance care planning, and recognising a need to discuss a patient’s future wishes and preferences. (Recommendations 10 to 11)

This phase describes activities relating to the initiation of a record as part of a DACP system. All patients with a terminal or life-limiting condition, regardless of disease type, should be offered an opportunity to create a DACP record. People with non-cancer conditions remain less likely to have advance care plans compared to those with cancer, despite making up about half of the palliative care caseload. Patients with shorter prognoses may find it easier to consider documenting wishes and preferences due to the predictability of their condition, whereas those with long-term, progressive disease face more challenges in planning for an uncertain future. There is an opportunity to use community-based resources, including online materials from governmental and charity organisations, to support patients prepare for advance care planning.

### Phase 3: Having conversations and documenting decisions (Recommendations 12 to 16)

This phase relates to the engagement of patients in the discussion of advance care planning information and the further digital documentation of preferences. DACP systems should ensure that plans are accessible, up-to-date and integrated across services, such as through summary care records. Patients and carers noted fragmented documentation across various services and often kept home-stored plans that were only accessible to healthcare professionals in person. Health professionals should proactively ask patients about any existing paper or electronic plans to facilitate their integration into digital systems. Assessing healthcare staff competency in advance care planning discussions with patients and subsequent digital documentation is essential. Those who see palliative patients less frequently may benefit from ongoing training updates to maintain their skills. Patients prefer discussing advance care planning with professionals who can dedicate time and understand their values, regardless of clinical background. Local guidelines on which services will access digitally created and stored advance care plans are needed, and this information should be conveyed to the patient so they are informed about who will see their information. Patients welcome the option to verify record accuracy, check who has accessed their information and prompt discussions when needed to reflect evolving care needs. Allowing patients and carers direct access to review and update their digitally stored advance care plans could help ensure that the information held is relevant and up-to-date. DACP systems should also offer secure, flexible login options (e.g. biometrics, security keys) to ensure ease of use without compromising security.

### Phase 4: Accessing, amending, actioning (Recommendations 17 to 19)

This phase is related to the iterative process of reviewing and updating records. DACP systems should enable filtering and summary views that ensure relevant information can be selected by different healthcare professionals for quick interpretation. Ambulance teams, for instance, value easily accessible summaries (e.g. relating to preferences around resuscitation) to make rapid care decisions in emergencies. Health professionals emphasised the need for DACP systems to prioritise and display the most recent information upfront, reducing the need for excessive scrolling through outdated entries. Patients preferred that holistic information be readily available to unfamiliar providers for personalised care. Hospital palliative care teams often cannot share advance care planning-related information with other hospital services, and patients have encountered difficulties when health professionals have not sought out information that has been shared across services. Systems should use notifications to flag patients with existing digitally stored advance care plans and prompt clinicians to review them, especially in acute settings such as emergency departments and specialty clinics. When working digitally the health professional should offer to share their computer screen with a patient (and their carer subject to patient permission) during advance care planning discussion or when a record of the discussion has been created. This will promote transparency as well as foster collaboration and patient empowerment.

### Phase 5: Using data to support evaluation and implementation (Recommendation 20)

This phase includes how DACP systems are evaluated and monitored, including, for example, quarterly reporting and providing feedback on outcomes achieved from DACP systems to professionals. The use of digital systems for advance care planning should be evaluated for their impact on care processes and findings shared with healthcare professionals and organisational management. In the UK, this aligns with the NHS Digital Health Technology Standard, which requires electronic health record developers to demonstrate how their products enhance care quality, patient outcomes and efficiency. Current measurement of DACP systems often relies on routine data extracted from the system itself, such as records of patient preferences and outcomes (e.g. preferred place of care or death). However, as patient wishes can change rapidly in their final days, this type of data may not fully capture the relevance or currency of care. More nuanced measures are needed, including tracking the frequency of records being reviewed (and potentially not altered) or updated. Additionally, gathering feedback from patients, carers and staff is essential to assess the real-world impact of DACP systems on care delivery. Patients and carers expressed interest in contributing to evaluations, hoping to collaborate with health professionals to develop better processes for future patients.

## Discussion

### Main findings

Data across the Optimal Care research programme has been synthesised to generate 20 recommendations to guide the design and future implementation of DACP systems. The Optimal Care programme represents the first comprehensive interdisciplinary research programme exploring DACP implementation. Multiple targets for the future development of DACP approaches were highlighted. Key themes within the recommendations were as follows: the critical role of stakeholder engagement in the design and implementation of DACP systems; the need for systems to be built on interoperable and accessible platforms that facilitate timely access for health and care professionals to up-to-date records; the need for DAPC systems to be embedded into routine workflows, with staff receiving appropriate training in communication around advance care planning; and the need for ongoing evaluation and continuous improvement to strengthen the evidence base underpinning DACP systems and to understand their impact on the delivery of care.

### Relation to existing research

Engaging patients and surrogates in advance care planning conversations is recognised as key for shared decision-making [48]. This research programme highlighted DACP systems as an acceptable approach to achieving this goal and for sharing relevant information about an individual with those providing care. DAPC systems are increasingly used internationally [17]. In England, the remit of DAPC systems is extending beyond documentation of future care preferences, with increasing emphasis on detailing broader aspects about an individual and supporting continuity of care across settings [3, 33, 49]. Despite their extending scope and implementation, our research highlighted low engagement with DACP systems, with most people dying without a digital record of their care preferences. We identified a slight increase in DACP records created early in 2020, aligned with increases in the number of patients with key aspects of advance care planning (e.g. resuscitation preferences) being recorded during the initial waves of the COVID-19 pandemic [50]. However, the number of DACP records created during this period remained substantially below the most conservative estimates of the number who might benefit from one [51].

Multiple barriers were highlighted that may hinder current DACP system implementation and influence professional engagement. Interoperability remains a persistent challenge and a priority for health and social care delivery in the UK [52]. Key providers involved in palliative care delivery, such as care homes and ambulance trusts, did not have access to information stored on DACP systems. This was due to a combination of different systems, devices, applications and organisations’ lack of ability to work together seamlessly to share and use data effectively. Ongoing efforts to improve interoperability of DACP systems include the development of clinical terminology and coding harmonisation [53], and proposals for changes in regulatory oversight [54]. Strong interoperability and information sharing are known to reduce duplication [55] and may reduce the need for repeated conversations for patients by ensuring their health information moves seamlessly across different care settings [56].

Alongside efforts to address the fragmented landscape of funding and governance for information technology systems in the UK, end-user-focused research should be prioritised. User-centred design and greater patient involvement are recognised as necessary steps for realising fully interoperable electronic health records in the UK NHS [57]. Across patients, carers and health and care professionals (including commissioners), this programme highlighted differing views on the purpose and intended impact of DACP systems, including who they are for and professionals’ responsibilities relating to their use. Changing work practices and evolving technology systems could create a context in which unintended consequences (e.g. care delivered that does not align with a person’s wishes due to inaccessible, outdated or inaccurate information) arise [58]. With complex information systems, there is ‘no silver bullet’ [59] (i.e. a single technology, methodology, or tool) that can solve all the challenges associated with their development and maintenance. However, at each phase of DACP system implementation, there are opportunities to enhance their relevance for health and care professionals working within local contexts. For instance, implementers should focus on planning, communication and continuous monitoring of health professionals’ roles and responsibilities when engaging with DACP systems. Defining roles and responsibilities will need to account for restrictions imposed by locally used clinical record systems. Alongside working to clarify roles and responsibilities, there is a need to develop processes for conveying benefits derived from DACP systems back to the professionals using them. This should include highlighting benefits for patient care and clinical practice compared to traditional ways of working [60, 61]. Furthermore, local evaluation of DACP systems is needed and should encompass aspects of care quality that capture the patient’s experience [62] as well as considering service outcomes, for example, the location where a person is cared for and dies [17]. There is also scope to explore audit and feedback approaches that emphasise action and interaction with DACP systems, which could be used to respond where low engagement with DACP systems is identified [63].

The evidence base underpinning advance care planning continues to be debated, partly due to gaps in the documentation, sharing and revising of a patient’s care preferences [64]. These are processes that can strengthened through DAPC systems, whose effective implementation can be guided by the recommendations developed through this research programme. However, the programme highlighted multiple potential tensions for the ongoing evolution of DACP systems. The content of DACP systems is moving away from traditional notions of advance care planning as part of end-of-life care, to encompass goals of care discussions for healthy people, and those with stable chronic illnesses [50, 65] including during early phases [66]. Furthermore, there is an increasing desire for patient access to DACP system content, which may lead to improved trust in recorded data, but this needs to be balanced with professionals’ views on how much information should be shared and maintaining the privacy and security of data [67].

This research programme provided novel insights into the views and experiences of patients and their carers on the role of DACP systems. Most DACP systems reported across the international literature do not enable patient access to their own record, despite increasing evidence of patient and carer support for access [68]. Key requirements for DACP systems for patients and carers generated from this research programme include (i) developing systems that enable patient (and potentially carer) review of documented preferences on DACP systems; (ii) the need to consider the role of existing online patient-facing resources to support preparation and readiness for advance care planning discussions [69–73]; (iii) drawing on community resources to raise awareness and support understanding around the role of DACP approaches and (iv) augmenting data recorded in records to better convey a holistic account of key patient characteristics, needs and preferences [39]. A further consideration around implementation is the ongoing work by community organisations providing education and support to people to develop advance care plans outside healthcare settings. Patient and carer participants highlighted ongoing engagement with community organisations, but also uncertainty about which health professional to engage in advance care planning discussions. This may result in challenges with sharing advance care plans with professionals or transferring the content of created plans to existing DACP systems.

### Future research

Effective DACP integration requires alignment with existing workflows, and future research should support the clarification of professionals’ roles, responsibilities and the intended impact of systems. The digital ecosystem is continually developing, and DACP systems need to respond to and fit within this context, for example, digital approaches to identify people with palliative care needs [74] which could be used to trigger DACP system interaction. A second key area for future research is focusing on patient and carer experiences and preferences for content in DACP systems, building trust in the systems and designing accessible, patient and potentially carer-friendly platforms. A third area is the testing and refinement of the conceptual model of digital advance care planning and recommendations derived from this research programme to determine how they influence system implementation and user engagement.

### Strengths and limitations

Evidence-informed recommendations for designing and evaluating DACP approaches have been developed through integrating five streams of research, incorporating multiple and diverse perspectives. Stakeholder engagement was extensive, including novel patient and carer perspectives on the role and design of DACP approaches. Health and care professional and policymaker perspectives were also gathered across multiple hospital and community settings, highlighting experiences and challenges in the use of existing digital approaches that support the documentation and sharing of advance care planning information. However, we highlight two study weaknesses. Complex systems such as DACP approaches may benefit from a systemwide exploration of their implementation. This programme retained a strong focus on the end-user experience which may have missed important aspects relating to the wider political, economic, regulatory and sociocultural context influencing their implementation. Furthermore, due to the limited uptake and engagement with DACP systems, our findings may reflect a minority view of professionals who are currently engaging with systems, highlighting the need for future research to validate stakeholder perspectives derived from this research programme for regions and populations where DACP systems are not currently in use but may be in the future. While we endeavoured to include diverse participants, the socio-economic, socio-cultural, and technological literacy of participants was not captured. This may limit the extent to which the generalisability of findings can be understood for contexts outside the UK.

## Conclusions

We have synthesised findings from multiple perspectives examined as part of the Optimal Care programme to generate evidence-based recommendations for the implementation of DACP systems, with relevance internationally. Interoperability remains a critical target of recommendations, particularly for seamless data sharing across all providers supporting palliative care delivery, including care homes and ambulance trusts. However, addressing technical barriers must be balanced with understanding diverse user needs, as differing views on system purpose and professional responsibilities risk unintended consequences, such as missed opportunities for goal-concordant care or misaligned care delivery. Furthermore, evaluation frameworks for the impact of DACP approaches on care quality require a shift in focus from service outcomes to patient-centred metrics.

## Data Availability

Deidentified transcripts generated and analysed during this research programme are available from the corresponding author on reasonable request.

## Appendix

Optimal Care recommendations to improve the design and implementation of digital advance care planning systems aligned with five phases of DACP and the domains of the NASSS framework

**Table:**

Phase 1: sociocultural, technical and structural prerequisites *Description: Aspects that need to be considered and in place ahead of a system being in place and used as part of routine care*
Recommendation 1. DACP systems should accommodate patient diversity—including varying disease trajectories and patient and carer backgrounds · Health professionals (WP3), patient and carer interviews (WP4) and workshop (WP5) data revealed that current DACP systems are more suited to people with a diagnosis of cancer. Systems design need to ensure relevance for people with conditions other than cancer, and those with multiple conditions, such as ensuring access for all relevant health services, integration across care pathways and the potential greater carer and proxy involvement in discussions and decision making documented on DACP systems. · Patient and carer (WP4) and workshop (WP5) participants suggested exploring the possibility of involving patients and carers from a range of backgrounds to inform culturally congruent content of DACP systems. *Aligned with NASSS Domain 1 - Health condition. *
Recommendation 2. Engagement with key stakeholders (i.e. health and care professionals, patients and carers) is essential to guide the content of DACP systems, alongside ensuring expectations, requirements, procedures and impact of the documentation and sharing of digital advance care plans are clear. Health professionals (WP3) reported the constant burden of training new staff including the increasing use of agency staff. General practitioners stated a need for guidance on roles and responsibilities of DACP in primary care and suggested this should be as brief and focused as feasible. Patient and carers (WP4) indicated that information should provide any health professional with enough knowledge about who they are as a person to treat them in a way that aligns with their wishes. Information should include · Holistic information about the person, · Information about preferred medical treatments and · Information about usual and preferred places of residence and preferred places of death. They emphasised the need for different electronic formats (Android, computer) and printed information. *Aligned with NASSS Domain 2 - Technology*
Recommendation 3. Organisations should prioritise and build DACP systems using platforms that support interconnectivity. This should ensure authorised health and social care workers across all relevant care settings can access digitally stored advance care planning documentation. Commissioner survey (WP1) data indicated that the implementation of digital systems for storing and sharing advance care plans varied significantly across services and geographical regions. The ability to view electronic advance care plans is limited, and most areas cannot share data with care homes or ambulance teams. Health professional survey (WP2) respondents reported that while digital systems supported the documentation of advance care plans, they were less helpful in sharing them. Health professional interviewees (WP3) stated critical gaps that exist with some DACP systems, including systems that do not share advance care planning information with ambulance teams or enabling access and sharing by care homes. Care home teams reported regularly engaging in and documenting advance care planning conversations with residents, but they could not view and access health services digital systems. If this were possible it would have allowed care home teams to check that information about their residents was accurate. Care home teams considered the content of care home systems superior and said that NHS health professionals would benefit from viewing it. They were aware of software that would join up with records in primary care, and that procurement was the responsibility of the general practice team or NHS service. *Aligned with NASSS Domain 2 - Technology*
Recommendation 4. Agreement on the purpose and intended impact of DACP systems should be established through consultation with all stakeholders involved in the care of patients with life-limiting illnesses, alongside patients and carers themselves. This engagement should guide the selection and measurement of DACP outcomes, which must be developed locally to account for geographical variations and differing approaches to DACP. Commissioner survey (WP1) respondents cited multiple intended impacts of DACP systems. Despite variation across geographical regions, there was broad agreement that these systems should facilitate the delivery of care following patient wishes and priorities. Health professional survey (WP2) data showed variation between health professional groups on the purpose of DACP systems. Respondents from care homes, hospices and ambulance teams were more likely than other groups to consider digital solutions to be distinct from other ways of working and were clear about their purpose. Health professional interview (WP3) data highlighted that palliative care teams, care homes and general practitioners need to be able to document, update and share quality advance care planning information; however, they also reported using verbal communication if situations are urgent and email or letters if they are not. Patient and carer interview (WP4) data indicated that patients valued aspects of planning, documenting and sharing their information. Patients with a digital advance care plan valued the documentation process even though they were unsure if it would be used to inform their care. Patient and carer interview (WP4) and Workshop data (WP5) highlighted the role of non-governmental organisations (e.g. Compassion in Dying, AGE UK, Macmillan) in supporting people to make advance care plans; therefore, these organisations should be involved in discussions about intended impacts and patient outcomes. Workshop data (WP5) also revealed multiple perspectives on which types of patients were eligible to have a shared digital advance care plan and what information the plan should contain. Variations in perspectives occurred between health professionals as well as patients and carers. *Aligned with NASSS Domain 3 - Value proposition*
Recommendation 5. The limitations of sharing information between services and regions should be clear to health professionals should this need to be communicated to patients. Health professional interview (WP3) participants indicated that it was not always known which other services could access shared information and in what format. Patient and carer interview (WP4) participants reported they were not sure who could see their data, some had presumed other services could access it at the point of need, then found this was not the case. *Aligned with NASSS Domain 4 – Adopters*
Recommendation 6. Organisations should learn from others who have developed activities to promote uptake and use (such as strengthening engagement or leadership and development of training). There is scope to identify and learn from creative solutions to promote uptake and use. Commissioner survey (WP1) respondents described the use of dashboard functionality to help stakeholders make informed decisions about patient care as well as to share learning across different organisations. Other activities reported included the development of a range of training resources, collaboration between regional care settings, engagement of senior management, strong clinical and IT leadership, and having an active communication strategy. Health professional interview (WP3) participants described how the integration of shared digital records with single point-of-access telephone line facilitated fast access to advance care planning information for both patients and other health professionals involved in patient care at the point of need. This data also revealed areas of good practice where the use of standard operating procedures in one hospital department had been rolled out to another. *Aligned with NASSS Domain 5 - Organisations *
Recommendation 7. Organisations should consider ways of embedding DACP into routine structures and processes (e.g. at admission, at discharge, multidisciplinary team meetings, and handovers). Health professional survey (WP2) data indicated a lower level of commitment to working together to build and sustain a community of practice around DACP systems among health professionals who did not work in hospice or ambulance teams. Health professional interview (WP3) data revealed that while high staff turnover can be a barrier to creating participation networks, this could be ameliorated by having key health professionals or managers in the organisation who drive forward engagement with digital systems for advance care planning as part of their designated role. *Aligned with NASSS Domain 5 - Organisations*
Recommendation 8. Training delivered within organisations should include data protection and legal or regulatory implications of methods of planning future care. Workshop (WP5) participants viewed data protection laws and safeguarding as crucial to consider in developing and using DACP systems. Participants, including health professionals, were not always clear on the legal or regulatory implications of different components and types of future planning (e.g. Advance Care Plans, Lasting Power of Attorney, Advanced Statements, Recommended Summary Plans for Emergency Care and Treatment, Advance decisions to refuse treatment (ADRT) and Do not attempt cardiopulmonary resuscitation (DNACPR or DNAR) decisions). *Aligned with NASSS Domain 6 - Wider System*
Recommendation 9. Initiatives that empower patients and families to engage in discussions about death and planning for end-of-life issues should be pursued. This may include the circulation of accessible resources (pamphlets, videos, website material) that offer guidance, alongside public engagement events such as Dying Matters Week. Patient and carer interview (WP4) discussions revealed that some patients could not remember if they had been approached about documenting preferences and some believed their health professionals considered it too early. Some reported that their cultural background had made discussions about their death difficult, and family members had avoided the topic when the patient wanted to have a conversation. Patient and carer interview (WP4) participants and workshop (WP5) participants talked about non-governmental organisations that had introduced them to thinking about advance care planning. These included AGE UK, Compassion in Dying, Macmillan Cancer Support and University of the Third Age. These organisations also produced a wide range of online resources as well as hosting events and one-to-one sessions. *Aligned with NASSS Domain 6 - Wider System*
Phase 2: Recognition of the clinical need for conversation and DACP (e.g. deterioration in condition, acute hospital admission or significant conversation), and recognising a need to discuss a patient’s future wishes and preferences (could be from a professional or patient). *Description: Initiating an advance care planning record on the digital system.*
Recommendation 10. Patients should be identified and offered the opportunity to document a digital advance care plan irrespective of disease type, with approaches explored to support the identification of non-cancer conditions. Commissioner survey (WP1) data revealed that only one-third of people who had died had any advance care planning information recorded. However, records for people with diseases other than cancer have increased since the start of the Covid-19 pandemic. Health professional interview (WP3) data suggested that patients without cancer make up most of the palliative care caseload, yet people with cancer are more likely to have a digital advance care plan. Patient and carer interviews (WP4) revealed that some participants without a clear clinical need had documented a digital advance care plan, whereas others with a long-term condition did not. Making choices appeared to be more straightforward for people with a short-term prognosis compared with a long-term progressive disease. Domestic life is more easily arranged around a patient for a short period than an unknown one. Patients often base wishes on what is realistic and available closer to death, so options are more likely to be known for cancer patients due to the more predictable nature of the disease. *Aligned with NASSS Domain 1 - Health condition*
Recommendation 11. There is scope to leverage existing community-based assets including organisations that provide in-person and patient-facing resources designed to support people to develop their own advance care plans. Existing patient-facing resources (including online resources) can be used to support preparation and readiness for advance care planning discussions. Patient and carer interview (WP4) participants spoke about the usefulness of information obtained from community-based organisations or downloaded from websites to inform their advance care planning decisions. Workshop (WP5) participants talked about governmental and charity organisations that are active in raising awareness and supporting advance care planning documentation and these services are useful in helping to prepare people for advance care planning and discussions. *Aligned with NASSS Domain 4 - Adopters *
Phase 3: having conversations and documenting decisions *Description: Engagement of patients in the discussion of advance care planning information. Digital documentation of preferences.*
Recommendation 12. Efforts should be made to ensure stored information is accessible through existing electronic medical record systems, for example, summary care records in England. Attempts should be made to ensure synchrony of information across record systems to ensure professionals can access exact and up-to-date patient preferences. Patient and carer interview (WP4) participants believed they haddifferent information stored in various services that could not be viewed or cross-checked by each service. *Aligned with NASSS Domain 2 - Technology*
Recommendation 13. Health professionals should elicit information from patients regarding any paper or electronic self-completed advance care plans they may hold and consider how to merge this information with the organisation’s digital system. Patient and carer interview (WP4) discussions about existing plans and components of plans (e.g. RESPECT, Advance directives) revealed that many had documents stored in different services and at home in electronic and paper formats. They often did not know where to take information stored only in the home, so it could be documented and shared with health professionals who need to see it to provide future care. *Aligned with NASSS Domain 4 - Adopters*
Recommendation 14. Undertaking a needs assessment of levels of staff competence in conducting advance care planning conversations with patients with life-limiting conditions can identify opportunities for delivering and tailoring training. There may also be scope to link competencies around communication and advance care planning within job descriptions for health and care professionals. Health professional interview (WP3) participants discussed the belief that some palliative care teams were better placed to carry out advance care planning discussions than others. While they were presumed to have more experience and time available than generalists, participants felt that any health or social care worker working with patients could and should undertake advance care planning discussions. Patient and carer interview (WP4) participants said they would prefer to discuss their advance care plan with a person who can dedicate enough time to discussing a range of options and likely outcomes and who can also get to know and understand the patient well. These characteristics were considered more important than a health professional’s clinical background. *Aligned with NASSS Domain 5 - Organisations*
Recommendation 15. There is a need for agreement and clarity on which services and settings can interact with a digitally stored advance care plan as soon as it is ready to share. This includes who can review, update and use the information to inform decision-making. Health professional survey (WP2) ambulance team respondents are more confident in accessing the digital system to view and use patient data than other professionals. Health professional interview (WP3) data indicated a general view that for digital systems to benefit patient care, all health professionals needed to contribute to the process of discussing, documenting and sharing their patients’ advance care plans. *Aligned with NASSS Domain 5 - Organisations*
Recommendation 16. Processes should be explored to ensure the content of DACP system records remain up-to-date and accurate. These processes may include, for example, options for patient and carer access to review the content of records or through discussion with patients. Different patients have varying preferences and comfort levels with technology, so access should allow flexibility with access and security (e.g. using either biometrics, security keys, social media credentials or passwords). Patient and carer interview (WP4) and workshop (WP5) patient and carer discussions included the recognition that documented wishes and preferences should be contemporaneous to support care delivery. Participants also acknowledged that the timing of subsequent discussions varied according to disease trajectory and patient resources. Patients welcomed the option to access their record to check for accuracy and a function to prompt or request a discussion with a health professional who could update their information. Patient and carer interview (WP4) participants indicated that viewing their records would be helpful for checking the accuracy of content. They would also value being able to check which professionals have accessed and edited their records. Participants did not want to constantly change or remember complex passwords. Logging in should be straightforward to facilitate continued use, and it must also be secure so that unauthorised users cannot access it. *Aligned with NASSS Domain 5 - Organisations*
Phase 4: accessing, amending, actioning. *Description: This includes the iterative process of reviewing and updating records*
Recommendation 17. Health professionals must ensure the advance care planning information they record is sufficient, but presentation in a summary or succinct form (e.g. highlighting resuscitation preference) may ensure accessibility and utility for different professional groups enabling them to provide care that aligns with patient wishes. Health professional survey (WP2) and interview (WP3) respondents working in ambulance teams said they valued the skill and input of other health professionals in documenting advance care plans and emphasised the importance of complete and up-to-date information they could find, read and interpret quickly to provide emergency care (such as information about resuscitation preferences). The ability to view the most up-to-date information quickly without the need to scroll or search, especially when using it to provide patient care in an emergency was shared across other health professional groups. Patient and carer interview (WP4) participants wanted holistic information to be the most readily available when receiving care from professionals who did not know them personally. *Aligned with NASSS Domain 2 - Technology*
Recommendation 18. Electronic patient record system notifications should flag patients with a digitally recorded advance care plan, and prompt clinicians to access and review. This should be across settings, including options for health and social care professionals working in relevant acute hospital specialties (e.g. emergency department, oncology, respiratory, cardiology) to have viewing access to DACP records created externally to the care setting at a minimum. Commissioner survey (WP1) highlighted most hospital palliative care teams could not share advance care planning information with other teams and services, even within the same hospital. Health professional interviews (WP3) suggested indicators on electronic systems were crucial for locating advance care planning information quickly; this was particularly important to Ambulance teams. Patient and carers (WP4) valued information sharing between different hospital services particularly if seen out of hours by professionals they do not know well. This was particularly key for patients with conditions needing input from different professionals. Patients had experienced instances where electronic information documented about them within one health service could not be viewed by another and anticipated similar challenges for DACP systems. *Aligned with NASSS Domain 2 - Technology*
Recommendation 19. While interacting with the digital systems, health professionals should work alongside patients (and their carers, if the patient permits) by offering to share the screen so patients feel part of their own planning. Patient and carer interview (WP4) participants expressed discomfort and detached from their plans and preferences when health professionals typed information they could not see and did not invite them to view. *Aligned with NASSS Domain 4 - Adopters*
Phase 5: Using data to support evaluation and implementation *Description: This could include, for example, quarterly reporting but also includes providing feedback on outcomes to professionals.*
Recommendation 20. The processes associated with the implementation of DACP systems and their effect on patient care should be measured and findings should be reported back to health professionals as well as organisation management. A range of appropriate methods of measurement may be needed to capture how DACP systems influence patient care and clinical decision making. Commissioner survey (WP1) data found that measuring the intended impact of DACP systems focussed on routinely collected data that counted the number of patients achieving a specific outcome (e.g. number of patients with a DACP system record at death, having a recorded preference for place of care or death). Several areas used feedback from staff, patients and families. Health professional interview (WP3) data revealed that counts of patient activity helped identify patients’ characteristics of who was less likely to have a record and could be used to help reduce inequality. However, wishes about the place of care or death can change quickly and frequently in a patient’s last days. Therefore, any outdated, digitally documented advance care planning data was deemed unsuitable for measurement, and additional measures are needed to assess how often records are being reviewed, updated and used to inform care. *Aligned with NASSS Domain 2 - Technology*

## Abbreviations

COVID-19: Coronavirus Disease 2019
DACP: Digital Advance Care Planning
EPaCCS: Electronic Palliative Care Coordination Systems
NASSS: Non-adoption, Abandonment, Scale-up, Spread and Sustainability
NHS: National Health Service
NoMAD: Normalisation MeAsure Development
UK: United Kingdom
WP: Work Package
WPs: Work Packages
